# A case of corneal epithelial downgrowth after penetrating keratoplasty treated by intracameral 5-fluorouracil injection and mitomycin C

**DOI:** 10.3205/oc000268

**Published:** 2026-03-11

**Authors:** Noel Hong-Kei Wat, Gillian Denise Ji-Yee Siu, Mei Sze Wu, Lee Wong

**Affiliations:** 1 Department of Ophthalmology, Caritas Medical Centre, Sham Shui Po, Hong Kong

**Keywords:** epithelial downgrowth, penetrating keratoplasty, 5-fluorouracil, mitomycin C, bullous keratopathy

## Abstract

**Background:**

Corneal epithelial downgrowth is an uncommon, yet potentially sight-threatening complication of intraocular surgery or trauma with poor prognosis. We hereby describe a case of epithelial downgrowth after penetrating keratoplasty which was treated by intracameral 5-fluorouracil injection.

**Case description:**

In 2019, penetrating keratoplasty was performed for a 69-year-old man with pseudophakic bullous keratopathy. Six months after penetrating keratoplasty, an endothelial opacity suspicious of epithelial downgrowth was noted. Two treatments of intracameral 5-fluorouracil injection were performed at the eighth and ninth month after penetrating keratoplasty, which allowed the epithelial downgrowth to become quiescent. A repeated penetrating keratoplasty with intracameral application of mitomycin C was subsequently performed without reactivation of the epithelial downgrowth.

**Conclusion:**

Intracameral 5-fluorouracil injection is effective in halting disease progression in epithelial downgrowth.

## Introduction

Corneal epithelial downgrowth is an uncommon, yet potentially sight-threatening complication of intraocular surgery or trauma. Historically, a number of invasive surgical options have been described including en-bloc excision, cryotherapy, and photocoagulation to treat this aggressive disease. We hereby describe a case of corneal epithelial downgrowth after penetrating keratoplasty which was controlled by intracameral 5-fluorouracil (5-FU) injection.

## Case description

A 69-year-old man presented to us in December 2017 with right eye persistent corneal edema four months after uneventful phacoemulsification was performed. He had a history of high myopia with myopic maculopathy and left eye amblyopia. According to the surgeon, pre-operative best corrected visual acuity (BCVA) of the right and left eyes were 0.2 and 0.1, respectively. There was no evidence of Fuchs’ endothelial dystrophy, and the pre-operative specular microscopy showed a cell count of 2,514 cells/mm^2^ and 2,134 cells/mm^2^ for the right and left eye, respectively. The right eye developed persistent corneal oedema with initial improvement in the first post-operative month with BCVA reaching 0.2. Post-operative anterior segment ocular coherence tomography (ASOCT) showed no Descemet’s membrane detachment. On presentation at our unit, BCVA of his right eye was 2/60 with diffuse corneal edema, likely due to corneal decompensation secondary to phacoemulsification. In view of corneal scarring from prolonged bullous keratopathy, an uneventful right eye penetrating keratoplasty (PKP) with donor graft size of 8.5 mm diameter to an 8.0 mm host trephine was performed in May 2019 and the post-operative recovery was smooth. Histopathology of the donor cornea showed loss of epithelium, an unremarkable Bowman’s layer; the Descemet’s membrane was not thickened and the endothelium was completely absent.

**Figure 1:**
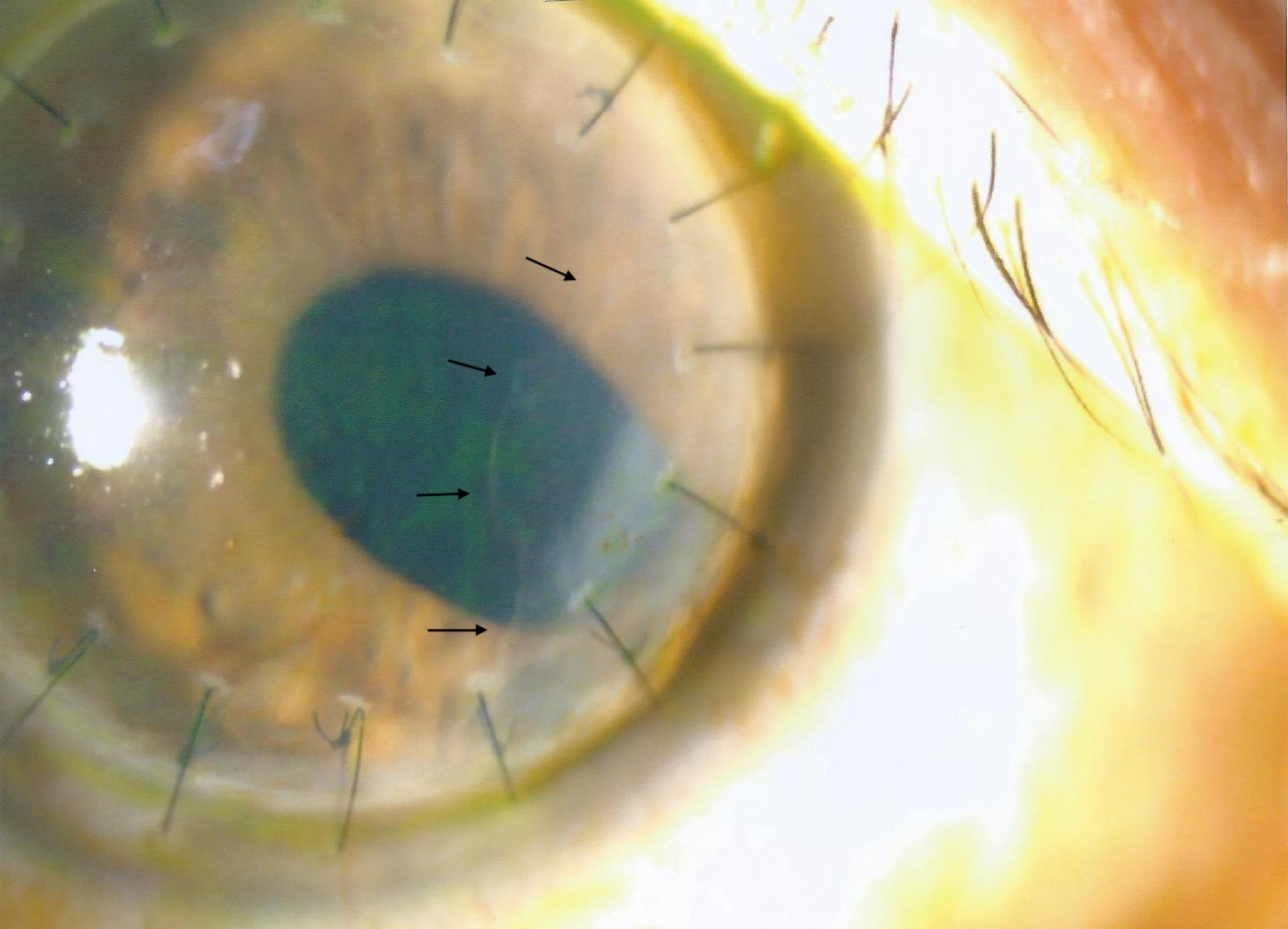
Nasal epithelial downgrowth (black arrows) seen at 6 months after PKP

At three months after PKP, his right eye BCVA was 0.5. However, at post-operative six-months, there was a nasal peripheral anterior synechiae (PAS) and a patch of endothelial opacity extending 1.5 mm into the centre from the graft edge, suspicious of epithelial downgrowth (Figure 1 (Fig. 1)). Furthermore, there was raised intraocular pressure (IOP) which was not well-controlled by maximal topical anti-glaucomatous drugs. In view of raised IOP and the epithelial downgrowth membrane, intracameral 5-FU 1 mg/0.1 mL was injected in January 2020 at post-PKP 8 months. 

**Figure 2:**
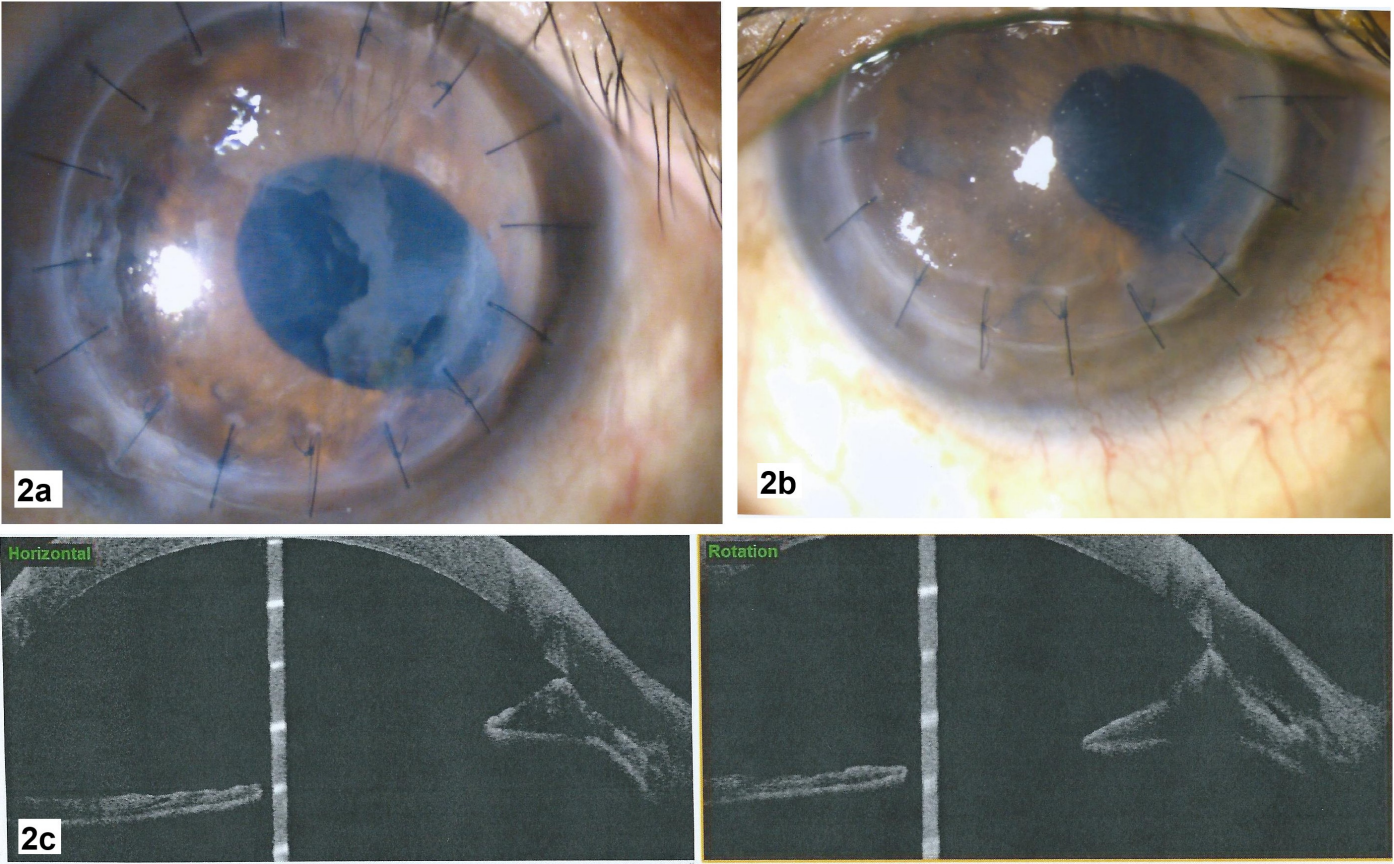
a: Progression of epithelial downgrowth 8 months after PKP, after first intracameral 5-FU injection. b: Mild corneal edema and persistent nasal PAS after regression of epithelial downgrowth membrane 4 months after second intracameral 5-FU injection. c: ASOCT showing persistent nasal PAS despite regression of epithelial downgrowth

Despite this initial treatment, the epithelial downgrowth membrane gradually grew to cover 70% of the graft, thereby obscuring the visual axis. There was also corectopia with ectropion uveae and graft edema (Figure 2a (Fig. 2)). The IOP was still not well controlled with maximal topical anti-glaucomatous drugs. Thus, a second intracameral 5-FU 1 mg/0.1 mL injection was performed one month later. On subsequent follow up, the epithelial downgrowth membrane had regressed (Figure 2b (Fig. 2)). ASOCT showed persistent nasal PAS despite clinical regression of the membrane (Figure 2c (Fig. 2)). 

At 10 months after the second intracameral 5-FU injection his right eye BCVA was 1/60. His right eye IOP was well-controlled, therefore anti-glaucomatous drugs were tapered to only Timolol 0.05% and Brimonidine tartrate 0.15%. However, the graft remained oedematous, possibly due to drug toxicity from the 5-FU and endothelial dysfunction from the epithelial downgrowth.

Later, an optical penetrating keratoplasty and intracameral application of mitomycin C (MMC) was performed in September 2022. A slightly smaller graft of 7.5 mm diameter to a 7.0 mm host trephine was done to avoid the area of previous epithelial downgrowth. Furthermore, a 0.2 mg/mL mitomycin-C-soaked cellulose sponge was applied directly to the area of focal PAS at 4 to 5 o’clock, which was presumed to be the area of previous epithelial downgrowth, for three minutes followed by thorough irrigation with balanced salt solution. This was done in hope to avoid any further reactivation of quiescent epithelial cells with the repeated penetrating keratoplasty. Microscopic examination of the bullous graft by the pathologist commented that the Descemet’s membrane was regular, the endothelium was completely absent and there was no inflammatory cell infiltration. The endothelium may have been undetectable due to scarcity of endothelial cells or due to injury to endothelial cells during formalin preservation and fixation for histopathological examination. The patient enjoyed a smooth early post-operative recovery with a clear graft and no recurrence of epithelial downgrowth.

**Figure 3:**
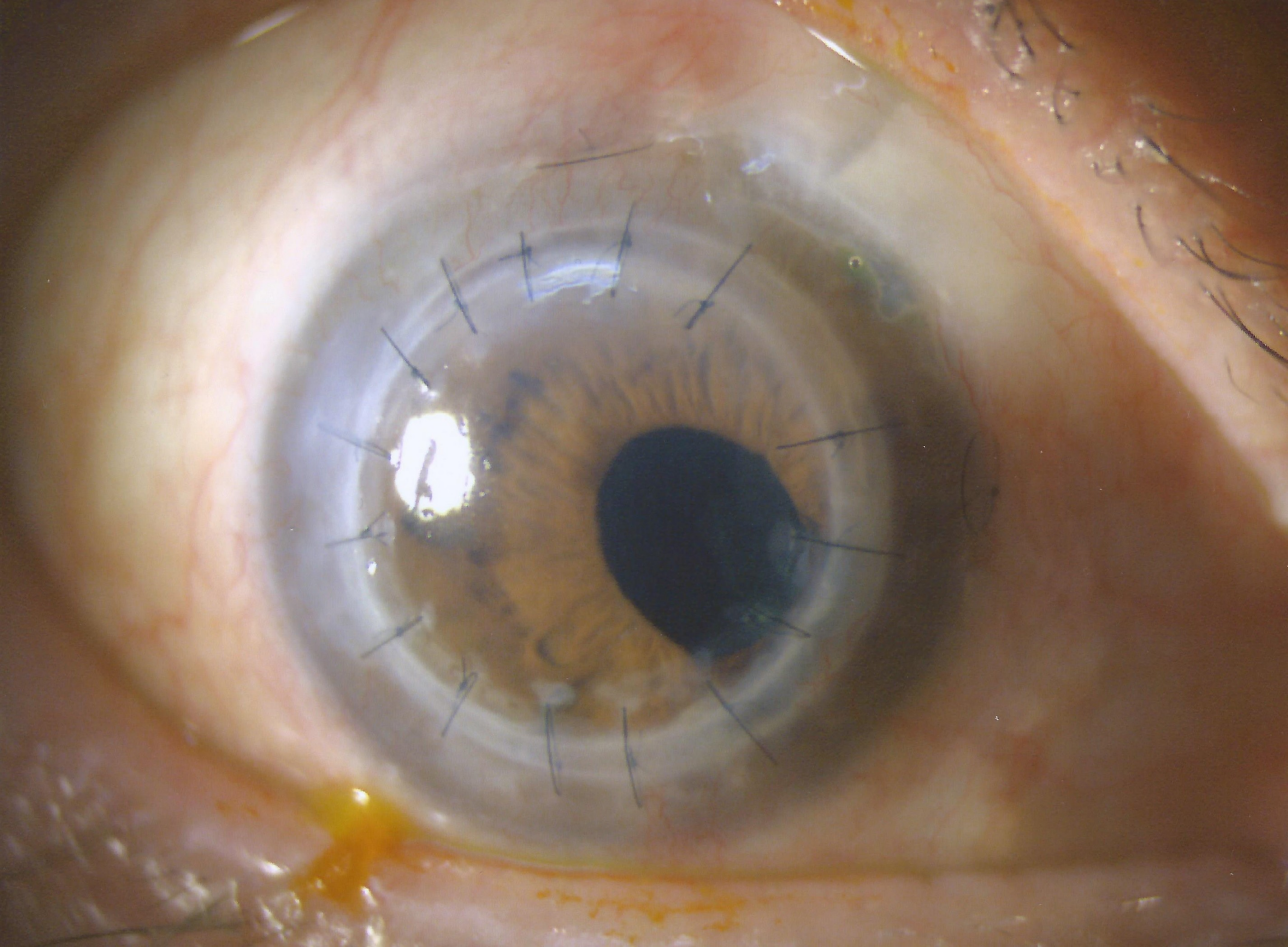
Clear corneal graft and superonasal trabeculectomy site with persistent nasal PAS at 1 year after repeated PKP

However, at post-operative three months after the second PKP, there was elevated IOP likely due to steroid response. The nasal focal PAS remained static and no further epithelial downgrowth membranes were seen on gonioscopy. Thus, trabeculectomy with mitomycin C was performed for the patient at five months after the second PKP and the IOP was well controlled thereafter. Additionally, right eye YAG capsulotomy was performed for the patient in view of posterior capsular opacification. Currently, at twelve months after the second PKP, the patient’s BCVA has improved to 0.4; the corneal graft remains clear and there has been no recurrence of the epithelial downgrowth (Figure 3 (Fig. 3)).

## Discussion

Although rare, epithelial downgrowth may be a devastating complication of intraocular surgery. It presents in two forms – the aggressive diffuse sheet-like form, as in this case, and the more benign local cystic form [1]. The proliferation of epithelial cells on the endothelium may lead to endothelial decompensation and corneal edema, or even intractable angle closure glaucoma when it proliferates over the trabecular meshwork. Management of this disease has previously required invasive surgical treatments including en bloc excision of the membrane, iridectomy, transcorneal cryotherapy, cautery, and photocoagulation [2]. Some patients may even require repeated penetrating keratoplasty. Furthermore, glaucoma drainage device implants may be needed for cases with secondary glaucoma [1]. Despite aggressive treatment, historically there has been poor prognosis with most eyes having a post-operative visual acuity of 20/200 or worse [2].

Use of antimetabolites as an alternative treatment for epithelial downgrowth was first suggested by Weiner et al. [2]. Case reports have employed doses of 5-FU ranging from 0.2 to 1 mg, and most required at least two sequential injections [3], [4], [5], [6], [7], [8]. In some cases, 5-FU was injected under air [8] or mixed with viscoelastic [3], [6] for better retention in the retrocorneal space. Some authors have even attempted viscodissection of the membranes [6] or have advocated for face-down positioning for 30 minutes postoperatively [8]. In the current case, direct injection of 5-FU into the anterior chamber with preoperative pilocarpine use yielded good results with no evidence of recurrence ten months after the second injection. Yet, we suggest refraining from epithelial membrane viscodissection, especially during the active phase of the disease, as it may release more epithelial cells into the anterior chamber and it is difficult to ensure complete membrane removal. Direct intracameral injection of 5-FU may already suffice.

Varying results after intracameral 5-FU injection have been reported. Some have suggested clinical evidence of regression of epithelial downgrowth without the need for further invasive surgery [6], [8]. Yet, others have found initial resolution with later recurrence of the epithelial downgrowth which required subsequent aggressive surgical treatment [3], [4], [5]. This may be because not all viable epithelial cells were eradicated with one injection. As 5-FU works by inhibiting actively proliferating cells, some clusters of epithelial cells may have been in the rest phase of division, causing them to be temporarily immune to 5-FU. As these cells transition to active cell division after the effect of 5-FU has waned, epithelial downgrowth may recur [3]. Hence, repeated injections of 5-FU may be more effective in targeting all epithelial cells in different phases of cell division to reduce recurrence. The re-treatment interval of 5-FU injection should be based on clinical response. 

The toxic effect of 5-FU on the corneal endothelium is controversial. Many have reported the need for repeated keratoplasty due to graft failure, despite no further progression of the epithelial downgrowth [3], [6]. However, Wong et al. reported no significant loss of endothelial cell count with specular microscopy in a case of epithelial downgrowth following Descemet stripping automated endothelial keratoplasty. In Wong et al.’s description, only one injection of 0.1 mL of 5-FU (0.4 mg/mL), a relatively lower dose compared to other case reports, was used which may account for a reduced toxic effect. Although repeated intraocular surgery and the effect of epithelial downgrowth on endothelial health may certainly play a role, the temporal relationship of corneal edema and graft failure after 5-FU injection cannot be ignored. Similarly, in the current case, bullous keratopathy developed despite quiescent epithelial downgrowth after 5-FU injection. 

A case report by Yu et al. has described direct intralesional injection of MMC into a cystic epithelial downgrowth for 5 minutes followed by aspiration. Although the authors used a much lower dosage of MMC at 0.0002 mg/mL, they did not report any recurrence of epithelial downgrowth. Yet it is important to keep in mind that generally the cystic form of epithelial downgrowth is more benign, hence the direct application of MMC to the lesion may still yield sustained efficacy despite the low dosage [9]. However, caution should be exercised when performing any injection or aspiration from a cystic form of epithelial downgrowth, as the cyst can liberate epithelial cells into the anterior chamber, causing the original benign form of epithelial downgrowth to become its aggressive sheet form [1], [10]. 

Another series of case reports by Assi et al. also described the application of 0.4 mg/mL of MMC soaked sponges to the chorioretina to prevent proliferative vitreoretinopathy in perforating and severe intraocular foreign body injuries. In this case series, no complications such as hypotony, iris atrophy or retinal necrosis were reported by the authors despite a higher dosage of MMC used [11]. Scleral application of MMC at a dosage of 0.2–0.4 mg/mL is commonly employed to augment trabeculectomy. We adopted a similar dosage of 0.2 mg/mL which did not cause any intraocular complications or recurrence of epithelial downgrowth. Furthermore, direct application with MMC-soaked sponges allowed for better intraoperative control to prevent inadvertent spread of MMC in the anterior chamber.

## Conclusion

Corneal epithelial downgrowth is an uncommon, yet potentially sight-threatening complication of intraocular surgery or trauma with poor prognosis. This case illustrates intracameral 5-FU injection as an effective and less invasive treatment option to halt the progression of epithelial downgrowth. Complications of this technique include the need for repeated injection, endothelial toxicity, and progression requiring more extensive treatment. Furthermore, intraocular application of MMC was useful in preventing recurrence of epithelial downgrowth on repeated penetrating keratoplasty. More experience and additional studies are needed to determine the optimal dosage, injection technique, and treatment interval of 5-FU as well as the optimal dosage of intraocular application of MMC. 

## Notes

### Competing interests

The authors declare that they have no competing interests.
